# Maternal Health Experiences, Depression, and Anxiety Among Refugees and Displaced Persons in Iraq: A Cross-Sectional Study

**DOI:** 10.7759/cureus.67645

**Published:** 2024-08-23

**Authors:** Hamdia Mirkhan Ahmed, Salih Ahmed Abdulla, Namir Al-Tawil, Kathryn Mishkin

**Affiliations:** 1 Physiotherapy Department, College of Health Sciences, Hawler Medical University, Erbil, IRQ; 2 Nursing Department, University of Kitab, Perde, IRQ; 3 Nursing Department, College of Nursing, Hawler Medical University, Erbil, IRQ; 4 Community Medicine Department, Hawler Medical University, Erbil, IRQ; 5 Research, Private Practice, Rockville, USA

**Keywords:** idp, refugee, syria, iraq, depression, anxiety, quality of life, maternal care

## Abstract

Background and objectives: Improving maternal health is one of the World Health Organization's (WHO) key priorities, grounded in a human rights approach and linked to efforts on universal health coverage. This study aimed to assess maternal health experiences among refugees and displaced women in Iraq during the COVID-19 pandemic.

Methods: A cross-sectional study was done on 1321 women of reproductive age living in four camps supervised by the Barzani Foundation Charity in the Kurdistan Region of Iraq from June to August 2021. Researchers developed a questionnaire, and the data was collected by four staff members working in the camps through direct interviews with women. The World Health Organization Brief (WHOQOL-BREF), generalized anxiety disorder seven-item (GAD-7), and Patient Health Questionnaire-9 (PHQ-9) scales were used to measure quality of life (QoL), anxiety, and depression. The frequency, percentage, percentile, and the Chi-square test were used for data analysis.

Results: The women's mean age (SD) was 31.9 (±7.7) years. Around two-thirds of women attended the camp’s healthcare facilities, and 16.4% attended the private facilities. Women mentioned the following as barriers to seeking health services: COVID-19 (387/29.3%), transportation (351/26.6%), lack of someone watching children (300/22.7%), and language (242/18.3%). The rate of high-level QoL among currently pregnant women (8/8.7%) was significantly (p = 0.002) less than the rate among nonpregnant women (297/24.2%). More than half (734/55.6%) of the women had mild depression, 247/18.7% had major depression, and 50/3.8% had severe major depression. Regarding anxiety, 580/43.9% had minimal anxiety, 467/35.4% had mild anxiety, 173/13.1% had moderate anxiety, and only 101/7.6% had severe anxiety.

Conclusions: Refugees and internal displaced women in Iraq have barriers to seeking maternal healthcare. They suffer from low QoL, depression, and anxiety. Factors affecting the quality and accessibility of maternal healthcare in the camps should be studied. Health policymakers have to consider its improvement.

## Introduction

Close to 20 million refugees continue to live in camps, globally. States of refuge often insist upon establishing camp settlements for reasons of public order and/or security, especially when confronted by a sudden influx of people fleeing conflict. Moreover, camps are often the most effective operational response to emergencies in conflict scenarios, as the United Nations High Commissioner for Refugees (UNHCR) recognizes [1].

Maternal health refers to women's health during pregnancy, childbirth, and postnatal. Each stage should support women and their babies to reach their full potential for health and well-being. More than half of maternal deaths occur in fragile and humanitarian settings. Improving maternal health is one of the World Health Organization's (WHO) key priorities, grounded in a human rights approach and linked to efforts on universal health coverage [2]. Decades of conflict, sanctions, and political instability have slowed Iraq's progress in reducing child and maternal mortality, but improving maternal and child health is a priority in the Ministry of Health’s strategic plans. Reproductive health services are improving after a decline following the 2003 conflict, but access to reliable data remains somewhat limited [3].

The Kurdistan Region of Iraq hosts 900,467 displaced individuals, comprising 631,174 internally displaced persons (IDPs) and 269,293 refugees from neighboring countries, especially from Syria. An estimated 110,000 Iraqi families, identified as IDPs, live across 33 camps within the main governorates: Erbil (41%), Duhok (40%), and Slemani (19%). Despite the support and respect from the Kurdistan government through the Barzani Charity Foundation, life in the camps is challenging, with acute food shortages and inadequate health services [4,5].

Pregnant women, women with young children, and these children themselves are among the most vulnerable groups within populations affected by conflict [6]. In crisis settings, access to health services is limited, which raises the risk of newborn mortality [7]. According to a 2018 UNICEF report, many women and newborns do not receive quality maternal and child care, even when they are able to access health facilities before, during, and after pregnancy and childbirth [7]. Refugee camps are believed to represent safe havens for forcibly displaced persons, but studies looking at refugees’ quality of life (QoL) in camps are few [8].

This study aimed to assess maternal health experiences among refugees and displaced women in Iraq. The specific objectives were to 1) display sociodemographic data of the sample study, 2) determine the self-reported QoL indicators, 3) identify different barriers to maternal healthcare reported, and 4) examine the frequency and severity of maternal mental health conditions (depression and anxiety) using the screening tools.

## Materials and methods

A cross-sectional study was completed with 1,321 women of reproductive age living in four camps (Hasan Sham, Debaga, Qushtappa, Darashakran) supervised by the Barzani Foundation Charity in the Kurdistan Region of Iraq. The camps were selected randomly from a total of 10 camps located in Erbil Governorate. Data collection took place during June to August 2021. A questionnaire format was developed, which includes sociodemographic data (nine questions), maternal health services (seven questions), barriers to seeking health services and antenatal care (ANC) (five questions), information regarding current pregnancy (11 questions), World Health Organization Brief (WHOQOL-BREF) for measuring the QoL, generalized anxiety disorder seven-item (GAD-7) anxiety scale, and Patient Health Questionnaire-9 (PHQ-9) Depression Scale. This questionnaire was not pretested and validated due to constraints in time and established questionnaires (three standard scales), which are often used in Middle Eastern countries.

The WHOQOL instruments were collaboratively developed in several centers worldwide and have been widely field-tested. The WHOQOL-BREF instrument comprises 26 items that measure the following broad domains: physical health, psychological health, social relationships, and environment. The is a shorter version of the original instrument (WHOQOL-100) that may be more convenient for use in large research studies or clinical trials. Scale scoring was done according to WHOQOL-BREF US guidelines [8]. Although the WHOQOL-BREF is a self-administered instrument, the data was collected through direct interviews with the participating women due to high rates of illiteracy. While this instrument is not validated in the Kurdistan Region and the Kurdish language, it is validated in the Arabic language in Jordan, which is also the formal language of Iraq [9].

The GAD-7 and PHQ-9 scales were used to assess for anxiety and depression, respectively. The GAD-7 has been consistently validated in low- and middle-income countries [10], including the Middle East. The scale consists of seven items, each corresponding to a symptom of anxiety such as nervousness, worry, restlessness, and difficulty relaxing. Respondents are asked to rate how often they have experienced each symptom over the past two weeks on a four-point scale (0 = not at all; 1 = several days; 2 = more than half the days; 3 = nearly every day). The total score ranges from zero to 21, with higher scores indicating more severe anxiety (0-4: minimal anxiety; 5-9: mild anxiety; 10-14: moderate anxiety; 15-21: severe anxiety. Similarly, the PHQ-9 has been used extensively in the Middle East Region, including Iraq, to assess for depression [11]. The questionnaire includes nine items, each corresponding to one of the core symptoms of depression such as loss of interest, feelings of hopelessness, changes in sleep or appetite, and thoughts of self-harm. Respondents are asked to rate how often they have experienced each symptom over the past two weeks on a four-point scale (0 = not at all; 1 = several days; 2 = more than half the days; 3 = nearly every day). The total score ranges from zero to 27, with higher scores indicating more severe depression (0-4: minimal depression; 5-9: mild depression; 10-14: moderate depression; 15-19: moderately severe depression; 20-27: severe depression).

In each camp, one staff of the Foundation was responsible for interviewing with the sample of the study in their tent. The researchers trained these staff on how to fill out the questionnaire. The researchers trained these staff to complete the questionnaire in one session and a group meeting. The training was provided by researchers in the office of the Barzani Charity Foundation, and each question in the questionnaire format was explained to them, and they were allowed to ask any question regarding the process of collecting data. Data were entered directly into MS Excel (Microsoft Corporation, Redmond, Washington, United States) on an electronic pad during interview.

We calculated our sample size using a single population proportion formula: prevalence = 0.5; 98% confidence interval, 0.03 margin of error; and 10% nonresponse rate (Table 1). We interviewed 1536 participants. After cleaning the data, those cases with missing data and those women above age 49 were excluded (188 women). In results, only 1,321 samples were considered for data analysis. We established three inclusion criteria: (1) participants must be women between the ages of 15 and 49 years, (2) identify as refugees or IDPs, and (3) be able to communicate in either Kurdish or Arabic language. We excluded participants with critical illness or who could not provide consent for their participation in the study.

The Scientific and Ethics Committee of the College of Health Sciences, Hawler Medical University approved the study proposal (number: Sc.E.C.4, 18/5/2020). Informed verbal consent was obtained from the study sample, and participants were free to withdraw from the study during data collection. In our community, people are often hesitant to provide written consent. They feel more comfortable with verbal agreements, as they believe that written consent might hold them accountable for any related issues.

Data was exported to IBM SPSS Statistics for Windows, Version 25 (Released 2017; IBM Corp., Armonk, New York, United States) and analyzed according to the study's objectives. Frequency, percentage, mean, median, percentile, and Chi-square test were used for data analysis. A p-value of <0.05 was considered statistically significant.

**Table 1:** Sample size calculation IDP: Internally displaced person

Name of camp	Type	No. of families	Population size	No. of females	Sample for margin of error at 5%	Sample after cleaning the data
Hasansham	IDP	2201	10060	5597	385	311
Darashakran	Refugee	2532	11511	Unknown	385	357
Dibaga	IDP	1418	7415	3802	396	311
Qoshtapa	Refugee	1956	8238	Unknown	370	342
Total					1536	1321

## Results

A total 1,321 women were included in the study, taken from four camps. Their mean age (SD) was 31.9 (7.7) years. The median was 31 years, and the age range was 16-49 years. The largest proportion (40.2%) were aged 20-29, and 37.1% were aged 30-39. More than half (52.9%) were Syrian; the rest were Iraqi women. More than half (53.2%) of the women were Kurds, and 45.8% were Arabs. Most (86%) of the women lived in the camp for over two years. The majority (70.2%) of the women were graduates of primary schools, and the income of 58.7% was less than 250, 000 ID per month. Regarding the number of persons living in one shelter, it was ≤5 women/shelter in 62.1% of the women. Only 4% of the women were working outside the home (Table 2).

**Table 2:** Sociodemographic characteristics of the study sample

	No.	(%)
Name of camp		
Hassan Sham	311	(23.5)
Debaga	311	(23.5)
Qushtappa	342	(25.9)
Darashakran	357	(27.0)
Age (years)		
<20	24	(1.8)
20-29	531	(40.2)
30-39	490	(37.1)
40-49	276	(20.9)
Nationality		
Iraqi	622	(47.1)
Syrian	699	(52.9)
Ethnic group		
Kurds	703	(53.2)
Arabs	605	(45.8)
Others	13	(1.0)
Years living in the camp		
<1 year	54	(4.1)
1-2 years	131	(9.9)
>2 years	1136	(86.0)
Educational level		
Illiterate	147	(11.1)
Primary school	927	(70.2)
High school	189	(14.3)
Institute and above	58	(4.4)
Income (Iraqi dinars/month)		
<250 000	775	(58.7)
250 000-500 000	455	(34.4)
500 001-750 000	56	(4.2)
750 001-1000 000	25	(1.9)
>1000 000	10	(0.8)
No. of persons living in one shelter		
≤5	820	(62.1)
>5	501	(37.9)
Working outside home		
Yes	53	(4.0)
No	1268	(96.0)
Total	1321	(100.0)

Around two-thirds of women attend the camp’s healthcare facilities, and only 16.4% attend the private facilities. It is evident in Table 2 that the women used to attend more than one healthcare facility. Regarding the distance to the camp’s healthcare facility, it was less than 15 minutes, according to 52.2% of the women’s responses, while the majority of the women who attend the private facilities take 31-45 minutes (Table 3).

**Table 3:** Maternal health services among the study sample

	No.	(%)
Where do you seek maternal healthcare?		
None	85	(6.4)
Camp	822	(62.2)
Camp + public facility	68	(5.1)
Camp + public facility + private facility	8	(0.6)
Camp + private facility	69	(5.2)
Public facility + private facility	11	(0.8)
Public facility	41	(3.1)
Private facility	217	(16.4)
Camp		
Yes	968	(73.3)
No	353	(26.7)
Public facility		
Yes	128	(9.7)
No	1193	(90.3)
Private facility		
Yes	305	(23.1)
No	1016	(76.9)
Distance to the camp health facility		
<15 minutes	689	(52.2)
15-30 minutes	65	(4.9)
31-45 minutes	130	(9.8)
>45 minutes	10	(0.8)
Not applicable	427	(32.3)
Distance to a public facility		
<15 minutes	63	(4.8)
15-30 minutes	49	(3.7)
31-45 minutes	32	(2.4)
>45 minutes	15	(1.1)
Not applicable	1162	(88.0)
Distance to a private facility		
<15 minutes	2	(0.2)
15-30 minutes	9	(0.7)
31-45 minutes	278	(21.0)
>45 minutes	18	(1.4)
Not applicable	1014	(76.8)
Total	1321	(100.0)

Women mentioned the following as barriers to seeking health services: language (18.3%), lack of someone watching children (22.7%), transportation (26.6%), and COVID-19 (29.3%). When asking pregnant ladies about the barriers to attending ANC services, around one-third (28.3%) mentioned that lack of transportation is a barrier, and 14.1% mentioned that they don’t know where to seek services (Table 4).

**Table 4:** Barriers to seeking health services and ANC by women ANC: Antenatal care (ANC) *Only 92 women were pregnant, and many didn’t provide information

	No.	(%)
Are language and/or cultural norms barriers for seeking health services?		
Yes	242	(18.3)
No	1079	(81.7)
Is the lack of someone watching children a barrier to seeking health services?		
Yes	300	(22.7)
No	1021	(77.3)
Is transportation a barrier to seeking health services?		
Yes	351	(26.6)
No	970	(73.4)
Are COVID-19 protocols a barrier to your seeking health services?		
Yes	387	(29.3)
No	934	(70.7)
Barriers to ANC (n = 92)*		
Not knowing where to seek services	13	(14.1)
Not feeling comfortable speaking language/cultural norms at health center	11	(12.0)
Not having transportation to go to the visits	26	(28.3)
Not having childcare for other children while you go to the visit	12	(13.0)
Others	5	(5.4)
Total	1321	(100.0)

It is evident in Table 4 that 92 women (7%) were pregnant. Around two-thirds (62%) of these pregnancies were planned, 19.6% of the women mentioned taking vitamins and folate before getting pregnant, and 65.2% took these vitamins during the current pregnancy. Around two-thirds (67.4%) of the women started the ANC visits during the first trimester. Half of the women claimed that they had gestational hypertension, and 42.4% had anemia. Almost all (97.8%) of the women mentioned that they were planning to breastfeed. Regarding the attitude of the health staff, 47.8% of the women mentioned that they were always treated with courtesy and respect by the doctors and nurses, and 47.8% mentioned that doctors and nurses always listened carefully to them. Most women mentioned that doctors and nurses explain things in a way they could understand. Most of the women (84.8%) had a history of deliveries before (Table 5).

**Table 5:** Current pregnancy information among pregnant women

	No.	(%)
Are you currently pregnant? (n = 1321)		
Yes	92	(7.0)
No	1229	(93.0)
Was your current pregnancy planned?		
Yes	57	(62.0)
No	35	(38.0)
Did you take vitamins and folate before getting pregnant?		
Yes	18	(19.6)
No	74	(80.4)
Are you taking vitamins/folate now?		
Yes	60	(65.2)
No	32	(34.8)
At which trimester did you start your antenatal care visit?		
First trimester	62	(67.4)
Second trimester	11	(12.0)
Third trimester	7	(7.6)
Did not attend till now	12	(13.0)
Any complications during this pregnancy?		
Gestational hypertension	46	(50.0)
Diabetes (DM)	5	(5.4)
Anemia	39	(42.4)
Bleeding	4	(4.3)
Others	15	(16.3)
Planning to breastfeed		
Yes	90	(97.8)
No	2	(2.2)
During this pregnancy, how often do doctors and nurses treat you with courtesy and respect?		
Never	2	(2.2)
Sometimes	15	(16.3)
Usually	31	(33.7)
Always	44	(47.8)
During this pregnancy, how often do doctors and nurses listen carefully to you?		
Never	2	(2.2)
Sometimes	11	(12.0)
Usually	35	(38.0)
Always	44	(47.8)
During this pregnancy, how often do doctors and nurses explain things a way you could understand?		
Never	2	(2.2)
Sometimes	17	(18.5)
Usually	34	(37.0)
Always	39	(42.4)
Have you ever delivered a child before?		
Yes	78	(84.8)
No	14	(15.2)
Total	92	(100.0)

The medians of the QoL domains were as follows: physical health (25), psychological health (18), social domain (12), and environmental health (24), as presented in Table 6.

**Table 6:** Scores of quality-of-life (QoL) domains of women

	Physical health (out of 35)	Psychological health (out of 30)	Social domain (out of 15)	Environmental health (out of 40)	Total QoL score (out of 120)
Mean	24.0	18.5	10.8	23.7	76.9
Median	25.0	18.0	12.0	24.0	77.0
SD	4.1	4.2	1.9	4.9	12.9
Minimum	11.0	8.0	3.0	8.0	35.0
Maximum	34.0	27.0	15.0	40.0	113.0

Regarding the total QoL score, the mean was 76.9, the median was 77, the first quartile (25th percentile) was 68, and the 75th percentile was 88 (Figure 1).

More than half (55.6%) of the women had mild depression, 18.7% had major depression, and 3.8% had severe major depression. Regarding anxiety, 43.9% had minimal anxiety, 35.4% had mild anxiety, and only 7.6% had severe anxiety (Table 7).

**Table 7:** Anxiety and depression among women *The total score ranges from 0 to 27. Cutoffs of 5, 10, 15, and 20 represent no depression, mild, major, and severe major depressive disorder, respectively. **Anxiety is categorized according to the following score: 0-4: minimal anxiety; 5-9: mild anxiety; 10-14: moderate anxiety; 15-21: severe anxiety

	No.	(%)
Depression		
No depression	290	(22.0)
Mild depression	734	(55.6)
Major depression	247	(18.7)
Severe major depression	50	(3.8)
Anxiety		
Minimal anxiety	580	(43.9)
Mild anxiety	467	(35.4)
Moderate anxiety	173	(13.1)
Severe anxiety	101	(7.6)
Total	1321	(100.0)

Women who scored less than the 25th percentile (25.3% of women) were considered as having a low total QoL, those in the interquartile area (between 25th and 75th percentiles) were considered as having average QoL scores (51.6%), and the rest (23.1%) who scored more than 88 out of 120 (75th percentile) were considered as having a high-level QoL as presented in Table 7. The table shows that 80.1% of the women in Debaga camp had high-level QoL compared with 1.1% and 2% of the women of Darashakran and Qushtappa camps, respectively (p < 0.001). Around half (47.3%) of the Iraqi women had high-level QoL compared with 1.6% of the Syrian women (p < 0.001). Significant (p = 0.005) association was detected with age, but it was not consistent, as high rates of high-level QoL were detected among those aged less than 20 (37.5%) and 40-49 (30.4%). The rate of high-level QoL among current pregnant women (8.7%) was significantly (p = 0.002) less than the rate among the nonpregnant women (24.2%), as presented in Table 8.

**Table 8:** Association between total QoL with camp name, nationality, age, and current pregnancy status QoL: Quality of life *Calculated by Chi-square test. **The score was categorized into low ≤ 68, average 69-88, and high > 88. ***Significant value

	Total QoL**				
	Low	Average	High	Total	p-value*
Camp name					
Hasan Sham	74 (23.8)	192 (61.7)	45 (14.5)	311 (100.0)	
Debaga	1 (0.3)	61 (19.6)	249 (80.1)	311 (100.0)	
Qushtappa	84 (24.6)	251 (73.4)	7 (2.0)	342 (100.0)	
Darashakran	175 (49.0)	178 (49.9)	4 (1.1)	357 (100.0)	<0.001***
Nationality					
Iraqi	75 (12.1)	253 (40.7)	294 (47.3)	622 (100.0)	
Syrian	259 (37.1)	429 (61.4)	11 (1.6)	699 (100.0)	<0.001***
Age					
<20	3 (12.5)	12 (50.0)	9 (37.5)	24 (100.0)	
20-29	130 (24.5)	294 (55.4)	107 (20.2)	531 (100.0)	
30-39	139 (28.4)	246 (50.2)	105 (21.4)	490 (100.0)	
40-49	62 (22.5)	130 (47.1)	84 (30.4)	276 (100.0)	0.005***
Currently pregnant?					
Yes	24 (26.1)	60 (65.2)	8 (8.7)	92 (100.0)	
No	310 (25.2)	622 (50.6)	297 (24.2)	1229 (100.0)	0.002***
Total	334 (25.3)	682 (51.6)	305 (23.1)	1321 (100.0)	

More than two-thirds (69.7%) of the women, who didn’t have depression had high-level QoL. This rate significantly decreases with increasing the severity of depression, reaching 2% among those with severe major depression (p < 0.001). Relatively the same pattern can be observed regarding anxiety where 46.7% of those with minimal anxiety had a high level of QoL, reaching 1% only in severe anxiety (p < 0.001), as presented in Table 9.

**Table 9:** Total QoL by anxiety and depression status QoL: Quality of life *Calculated by Chi-square test. **The score was categorized into low ≤ 68, average 69-88, and high > 88. ***Significant value

	Total QoL**				
	Low	Average	High	Total	p-value*
Depression level					
No depression	19 (6.6)	69 (23.8)	202 (69.7)	290 (100.0)	
Mild depression	179 (24.4)	455 (62.0)	100 (13.6)	734 (100.0)	
Major depression	128 (51.8)	117 (47.4)	2 (0.8)	247 (100.0)	
Severe major depression	8 (16.0)	41 (82.0)	1 (2.0)	50 (100.0)	<0.001***
Anxiety level					
Minimal anxiety	62 (10.7)	247 (42.6)	271 (46.7)	580 (100.0)	
Mild anxiety	149 (31.9)	292 (62.5)	26 (5.6)	467 (100.0)	
Moderate anxiety	93 (53.8)	73 (42.2)	7 (4.0)	173 (100.0)	
Severe anxiety	30 (29.7)	70 (69.3)	1 (1.0)	101 (100.0)	<0.001***
Total	334 (25.3)	682 (51.6)	305 (23.1)	1321 (100.0)	

Table 10 shows that there were significant association between being pregnant and having depression and anxiety. Another highly significant associated factors with having depression and anxiety were camp, age, nationality, ethnic group, and income (Table 11).

**Table 10:** Association between depression and anxiety with pregnancy *Chi-square was used. **Significant value

	Are you currently pregnant?		p-value*
	Yes	No	
Depression			
Yes	83 (90.2)	9 (9.8)	0.003**
No	984 (77.1)	281 (22.9)	
Anxiety			
Yes	54 (58.7)	38 (41.3)	0.602
No	687 (55.9)	542 (44.1)	

**Table 11:** Associated factors with depression and anxiety among the study sample *Chi-square was used. **Significant value

	Depression		p-value	Anxiety		p-value*
	Yes	No		Yes	No	
Name of camp						
Hassan Sham	298 (95.8)	13 (4.2)	<0.001**	270 (86.8)	41 (13.2)	<0.001**
Debaga	88 (28.3)	223 (71.7)		5 (1.6)	306 (98.4)	
Qushtappa	326 (95.3)	16 (4.7)		197 (57.6)	145 (42.4)	
Darashakran	319 (89.4)	38 (10.6)		269 (75.4)	88 (24.6)	
Age (years)						
<20	15 (62.5)	9 (37.5)	0.024**	7 (29.2)	17 (70.8)	0.003**
20-29	413 (77.8)	118 (22.2)		304 (57.3)	227 (42.7)	
30-39	399 (81.4)	91 (18.6)		292 (59.6)	198 (40.0)	
40-49	204 (73.9)	72 (26.1)		138 (50.0)	138 (50)	
Nationality					347	
Iraqi	386 (62.1)	236 (37.9)	<0.001**	275 (44.2)	347 (55.8)	< 0.001**
Syrian	645 (92.3)	54 (7.7)		466 (66.7)	233 (33.3)	
Ethnic group						
Kurds	644 (91.6)	59 (8.4)	<0.001**	455 (64.7)	248 (35.3)	< 0.001**
Arabs	374 (61.8)	231 (38.2)		276 (45.6)	329 (54.4)	
Others	13 (100)	0 (0)		10 (76.9)	3 (23.1)	
Income (Iraqi dinars/month)						
<250 000	536 (69.2)	239 (30.8)	<0.001**	390 (50.3)	385 (49.7)	< 0.001**
250 000-500 000	414 (91.0)	41 (9.0)		289 (63.5)	166 (36.5)	
500 001-750 000	50 (89.3)	6 (10.7)		39 (69.6)	17 (30.4)	
750 001-1000 000	23 (92)	2 (8.0)		19 (76.0)	6 (24.0)	
>1000 000	8 (80)	2 (20.0)		4 (40.0)	6 (60.0)	

## Discussion

This study presents the experiences of women of reproductive age regarding maternal care services who were living in the camps due to conflicts in Iraq and Syria. The majority used the maternal healthcare facility in the camp. Many factors, such as low income and distance from private clinics, were barriers to using higher-quality maternity care outside the camp. Miscommunication was another woman’s experience during health-seeking care. Low QoL, depression, and anxiety were more common among refugees than IDPs.

These results are congruent with another cross-sectional study conducted at four Syrian refugee camps in the Kurdistan Region-Erbil in 2016, on 470 newly delivered women who had at least one ANC visit during pregnancy, with 73.6% having adequate ANC visits. About 64% of the women visited public and private health sectors. There was no statistically significant association between the mothers' age and education with the utilization of adequate ANC [11]. In general, providing primary healthcare, including maternal care, is a strong point of the health system in the Kurdistan Region of Iraq. The results of another cross-sectional study, which has been conducted to assess the knowledge and practices of 103 women attended to ANC at Arbat camps in Sulaimanya, Iraq are witnesses of that. The results show that most pregnant women have very good knowledge about ANC except for performing oral health hygiene and taking medicine during pregnancy [12]. It is worth mentioning that health services in the Kurdistan Region of Iraq are mainly provided by the Ministry of Health, free of charge. Syrian refugees and IDPs have free access to health services, including consultations, medicines, laboratory tests, screening, surgeries, and maternal and child care in public hospitals and primary healthcare centers [13]. However, the distance of public and private services and transportation played a role in accessing them by refugees and IDPs.

It is evident that there are some barriers to seeking or accessing healthcare in conflict areas and camps. Factors such as financial constraints, language barriers, and discrimination were contributed to limited healthcare access among refugees, asylum seekers, undocumented migrants, and IDPs during COVID-19 pandemic [14]. In the present study, language, cultural factors, lack of supportive persons, and transportation were the main barriers. Some of these barriers were presented in other studies that were reviewed in a scoping review. Ten groups of factors facilitating and/or limiting access to sexual and reproductive healthcare among migrant, internally displaced, asylum seeking, and refugee women emerged from the synthesis of the retained articles. The main barriers were lack of knowledge about services, cultural unacceptability, financial inaccessibility, and language barriers between patients and healthcare providers. Facilitators included mobile applications for translation and telehealth consultations, patients with a wide availability of information sources, health promotion representatives, and healthcare providers trained in cultural sensitivity, communication, and person-centered care [15]. In a qualitative study in Somalia among IDPs in Mogadishu, various factors were identified as barriers to the utilization of maternal and child health services, including low socioeconomic, lack of family support, transportation challenges, poor functional services, and negative experiences [16]. In a mixed-method study among IDP camps on 587 females, the most common reason for not accessing ANC and not seeking postpartum care was not having enough money and the lack of finances [17]. Although the sample of the present study did not mention this reason, results show that the majority of the women had very low incomes, which may affect their transportation expenses to health facilities.

COVID-19 was another barrier among the present study's sample for accessing healthcare, which is similar to the results of other studies, a critical ethnographic study on 27 women who were refugees. Refugee women were at high risk of experiencing add-on stressors due to isolation, difficulty in accessing healthcare, COVID-19-related restrictions in hospitals, limited follow-up care, limited social support, financial challenges, and compromised nutrition [14,18].

Although in the present study, only refugees and IDPs inside camps were studied to measure their QoL, a report from World Bank Group, which analyzed the case of Syrian refugees living in Jordan, confirms that the refugees' QOL is low likewise, the results present that refugees living out of camps enjoy relatively higher QOL than those living in camps [8]. In the present study, results indicate that Syrian refugees had lower QoL compared to IDPs. Results of a study done on 523 Syrian refugee women in the host communities in Jordan shows that the general health and HRQoL of Syrian refugee women were low compared to the findings of other studies of Arab women. There is a relationship between low socioeconomic status and poor HRQoL. Several factors, such as years of marriage, age at marriage, the number of children, violence, antenatal care, and family income, affected the women’s general health. The association of these factors was not studied in the present study [19].

Pregnancy was an associated factor with low QoL among refugees and IDPs in the present study. These results are different from the results of a cross-sectional study done on the QoL of 319 pregnant women who was recruited from two maternal health clinics at the Al-Zaatari refugee camp in Jordan showed that despite the acceptable level of satisfaction with QOL among pregnant Syrian refugees women in the study, women were least satisfied with their physical health [20]. 

Generally, anxiety, depression, and suicidal thoughts/attempts are prevalent among Iraqi women post-conflict [21]. If we add the challenges of living in camps to the background life in Iraq, the presence of depression and anxiety is expected. The results of the present study confirm the presence of anxiety and depression among women living in the camps, which had a highly significant association with low QoL. Age, nationality, ethnic group, camp, monthly income, and being pregnant were associated factors with having anxiety and depression. The results of another study done on 494 married couples who were Syrian Kurdish refugees in the Kurdistan Region of Iraq are consistent with the present research results. Approximately two-thirds of them experienced probable depression, which was associated with gender, age, time spent in the camp, and the number of traumatic event types [22]. The results of another study show that the rates of perinatal depressive symptoms risk among internally displaced Yazidi pregnant and postpartum women are higher than the general Kurdish-speaking population in Iraq (28.4%) [23].

Depression at different levels has also been seen among women living in refugee camps in Duhok City of the Kurdistan Region, which is congruent with the results of the present study [24]. A study of mental health status among Syrian refugee women living in Jordan shows high levels of exposure to traumatic events. The study findings also demonstrated a significantly high but unspoken burden of mental health problems among Syrian refugee women living outside camps [25]. Being a woman was a risk factor for getting major depressive disorders among Syrian refugees in Greece [26]. Living conditions are an important contextual factor affecting refugees’ mental health. Refugees living in camps and in urban settings may have different mental health needs. A field survey was conducted among 1,470 refugees living in camps and urban settings in Turkey. Both post-traumatic stress disorder (PTSD) and depression were more common in urban settings than in camps [27].

Limitations

Although the inclusion of four camps of refugees and IDPs and a large sample size in the present study are strength points, involving a small group of pregnant women and not including the characteristics of maternal health services in each camp are limitations of the present study. Not conducting pretesting and validation of the whole questionnaire was another limitation which can be considered for further studies.

## Conclusions

Although a large proportion of Syrian refugees and Iraqi IDP women are seeking maternal healthcare in their camps, there are barriers to access and well utilization of the services. The QoL of Syrian refugee women is worse than Iraqi IDPs. Refugees and IDP women are suffering from anxiety and depression and are associated with sociodemographic and obstetrical factors. Providing maternal healthcare services with specific strategies and enrichment with mental healthcare have to be prioritized by health policymakers in Iraq and all conflict areas in the world. Easy access to maternal healthcare will improve health outcomes and QoL and reduce the mental health burden among refugees and IDPs.

## References

[1] Donnelly ER, Muthiah V (2019). Protecting women and girls in refugee camps: states' obligations under international law. https://eprints.lse.ac.uk/110299/1/Muthiah_protecting_women_and_girls_published.pdf.

[2] (2024). Maternal health. https://www.who.int/health-topics/maternal-health.

[3] (2024). Maternal, newborn, child and adolescent health. https://www.emro.who.int/iraq/priority-areas/maternal-newborn-child-and-adolescent-health.html.

[4] Kurdistan Region (2024). Kurdistan region: a beacon of hope for displaced persons and refugees. https://gov.krd/dmi-en/activities/news-and-press-releases/2024/march/kurdistan-region-a-beacon-of-hope-for-displaced-persons-and-refugees/.

[5] (2024). Syrian crisis Iraq impact assessment: IOM. https://www.iom.int/news/syrian-crisis-iraq-impact-assessment-iom.

[6] Nidzvetska S, Rodriguez-Llanes JM, Aujoulat I, Gil Cuesta J, Tappis H, van Loenhout JA, Guha-Sapir D (2017). Maternal and child health of internally displaced persons in Ukraine: a qualitative study. Int J Environ Res Public Health.

[7] Casey SE (2015). Evaluations of reproductive health programs in humanitarian settings: a systematic review. Confl Health.

[8] Obi CT (2021). The impact of living arrangements (in-camp versus out-of-camp) on the quality of life. Case study of Syrian refugees in Jordan. World Bank Working Paper.

[9] Dalky HF, Meininger JC, Al-Ali NM (2017). The reliability and validity of the Arabic World Health Organization quality of life-BREF instrument among family caregivers of relatives with psychiatric illnesses in Jordan. J Nurs Res.

[10] Policastro F, Rossi A, Sulaiman HM, Taib NI (2023). Adaptation, validity, and reliability of the Patient Health Questionnaire (PHQ-9) in the Kurdistan Region of Iraq. Healthcare (Basel).

[11] Fareed SM, Ismail KH (2019). Utilization of antenatal care services in Syrian refugee camps in Erbil, Iraq. Zanco J Med Sci.

[12] Abass LA (2018). Assessment of knowledge and practices of internally displaced pregnant women attending to antenatal clinic center at Arbat camp in Sulaimani, Kurdistan Region of Iraq. KJAR.

[13] (2024). Refugees and internally displaced populations. https://extranet.who.int/kobe_centre/sites/default/files/WHO%20Guidance_Research%20Methods_Health%20EDRM_2021_Chapter-5.3.pdf.

[14] El Arab RA, Somerville J, Abuadas FH, Rubinat-Arnaldo E, Sagbakken M (2023). Health and well-being of refugees, asylum seekers, undocumented migrants, and internally displaced persons under COVID-19: a scoping review. Front Public Health.

[15] Sawadogo PM, Sia D, Onadja Y (2023). Barriers and facilitators of access to sexual and reproductive health services among migrant, internally displaced, asylum seeking and refugee women: a scoping review. PLoS One.

[16] Mohamed AA, Bocher T, Magan MA (2021). Experiences from the field: a qualitative study exploring barriers to maternal and child health service utilization in IDP settings Somalia. International Journal of Women's Health.

[17] Mohamed AA, Bocher T, Magan MA, Omar A, Mutai O, Mohamoud SA, Omer M (2021). Experiences from the field: a qualitative study exploring barriers to maternal and child health service utilization in IDP settings Somalia. Int J Womens Health.

[18] Hirani SA, Wagner J (2022). Impact of COVID-19 on women who are refugees and mothering: a critical ethnographic study. Glob Qual Nurs Res.

[19] Nabolsi M, Safadi R, Sun C (2020). The health-related quality of life of Syrian refugee women in their reproductive age. PeerJ.

[20] Alnuaimi K, Alshraifeen A, Aljaraedah H (2022). Factors influencing quality of life among Syrian refugees pregnant women in Jordan: a cross-sectional study. Heliyon.

[21] Lafta RK, Merza AK (2021). Women's mental health in Iraq post-conflict. Med Confl Surviv.

[22] Mahmood HN, Ibrahim H, Goessmann K, Ismail AA, Neuner F (2019). Post-traumatic stress disorder and depression among Syrian refugees residing in the Kurdistan region of Iraq. Confl Health.

[23] Seidi PA, Abas NQ, Jaff D (2022). Assessment of perinatal depression risk among internally displaced Yazidi women in Iraq: a descriptive cross-sectional study. BMC Pregnancy Childbirth.

[24] Abas NQ (2018). Gender differences in depression levels at refugee camps in Duhok City, Kurdistan Region of Iraq. JUG.

[25] Kheirallah KA, Al-Zureikat SH, Al-Mistarehi AH (2022). The association of conflict-related trauma with markers of mental health among Syrian refugee women: the role of social support and post-traumatic growth. Int J Womens Health.

[26] Poole DN, Hedt-Gauthier B, Liao S, Raymond NA, Bärnighausen T (2018). Major depressive disorder prevalence and risk factors among Syrian asylum seekers in Greece. BMC Public Health.

[27] Isik E, Sismanlar SG, Tekeli-Yesil S (2024). PTSD, depression, and migration-related experiences among Syrian refugees living in camp vs urban settings. Transcult Psychiatry.

